# CDK5RAP2 loss-of-function causes premature cell senescence via the GSK3β/β-catenin-WIP1 pathway

**DOI:** 10.1038/s41419-021-04457-2

**Published:** 2021-12-20

**Authors:** Xidi Wang, Patrick Sipila, Zizhen Si, Jesusa L. Rosales, Xu Gao, Ki-Young Lee

**Affiliations:** 1 Department of Cell Biology & Anatomy, Arnie Charbonneau Cancer and Alberta Children’s Hospital Research Institutes, Cumming School of Medicine, University of Calgary, Calgary, AB Canada; 2 Department of Biochemistry & Molecular Biology, Harbin Medical University, Harbin, China

**Keywords:** Senescence, Diseases

## Abstract

Developmental disorders characterized by small body size have been linked to CDK5RAP2 loss-of-function mutations, but the mechanisms underlying which remain obscure. Here, we demonstrate that knocking down CDK5RAP2 in human fibroblasts triggers premature cell senescence that is recapitulated in *Cdk5rap2*^*an/an*^ mouse embryonic fibroblasts and embryos, which exhibit reduced body weight and size, and increased senescence-associated (SA)-β-gal staining compared to *Cdk5rap2*^+/+^ and *Cdk5rap2*^*+/an*^ embryos. Interestingly, CDK5RAP2-knockdown human fibroblasts show increased p53 Ser15 phosphorylation that does not correlate with activation of p53 kinases, but rather correlates with decreased level of the p53 phosphatase, WIP1. Ectopic WIP1 expression reverses the senescent phenotype in CDK5RAP2-knockdown cells, indicating that senescence in these cells is linked to WIP1 downregulation. CDK5RAP2 interacts with GSK3β, causing increased inhibitory GSK3β Ser9 phosphorylation and inhibiting the activity of GSK3β, which phosphorylates β-catenin, tagging β-catenin for degradation. Thus, loss of CDK5RAP2 decreases GSK3β Ser9 phosphorylation and increases GSK3β activity, reducing nuclear β-catenin, which affects the expression of NF-κB target genes such as WIP1. Consequently, loss of CDK5RAP2 or β-catenin causes WIP1 downregulation. Inhibition of GSK3β activity restores β-catenin and WIP1 levels in CDK5RAP2-knockdown cells, reducing p53 Ser15 phosphorylation and preventing senescence in these cells. Conversely, inhibition of WIP1 activity increases p53 Ser15 phosphorylation and senescence in CDK5RAP2-depleted cells lacking GSK3β activity. These findings indicate that loss of CDK5RAP2 promotes premature cell senescence through GSK3β/β-catenin downregulation of WIP1. Premature cell senescence may contribute to reduced body size associated with CDK5RAP2 loss-of-function.

## Introduction

CDK5RAP2 was identified based on its ability to interact with the Cdk5 regulatory subunit, p35 [1]. CDK5RAP2 localizes to the centrosome in a dynein-dependent manner [2] and regulates centriole engagement and centrosome cohesion during the cell cycle [3–5]. CDK5RAP2 moved into the spotlight as loss-of-function mutations cause primary microcephaly, an autosomal recessive neurodevelopmental disorder characterized by the small brain and cognitive deficit [6, 7]. In fact, CDK5RAP2 is most abundant at the luminal surface of the brain’s ventricular zone, particularly in cells lining the ventricular wall where the neural stem and progenitor cells reside. However, since CDK5RAP2 is also expressed in other tissues, it is not surprising that loss-of-function mutations are further associated with other developmental disorders [8]; e.g., primordial dwarfism [9, 10] and Seckel syndrome [11]. However, the molecular mechanisms by which CDK5RAP2 loss-of-function mutations cause these developmental disorders to remain elusive.

Cellular senescence is a state of stable cell cycle arrest where cells remain viable and metabolically active [12]. It is characterized by increased SA-β-gal activity [13], senescence-associated heterochromatin foci (SAHF) formation [14], and morphological transformation [15]. Senescence is triggered by cellular stresses [16–19]. For example, activated oncogenes such as H-RAS^*G12V*^ and B-RAF^*V600E*^ induce senescence by evoking sustained anti-proliferative response [20], which acts as an initial barrier in preventing normal cell transformation into malignant cells. H-RAS^*G12V*^-induced senescence is associated with DNA damage foci accumulation and p53 kinase activation, suggesting that aberrant oncogene activation induces DNA damage response (DDR) [20, 21]. Phosphorylation of the p53 tumor suppressor protein at Ser15 by p53 kinases such as ATM, Chk1, and/or Chk2 is one of the key events in p53-associated cell senescence [22, 23]. Apparently, p53 Ser15 phosphorylation stabilizes p53 by inhibiting its interaction with Mdm2, an E3 ubiquitin ligase that catalyzes polyubiquitination, subsequently inducing proteasome degradation of p53 [24]. However, p53 Ser15 phosphorylation is also required for p53 activation [25, 26], that blocks cell cycle progression by inducing p21^*CIP1*^ expression [27]. Aside from phosphorylation by kinases, p53 is also regulated by phosphatases such as WIP1. In fact, WIP1 has been associated with p53-mediated cell senescence [28, 29]. For example, hematopoietic stem cells (HSC) from WIP1^−/−^ mice exhibit senescent phenotypes, impairing repopulating activity [29]. MEFs [29] and mesenchymal stem cells [30] from WIP1^−/−^ mice also undergo premature senescence. In primary chondrocytes, reduced WIP1 is associated with senescent phenotype, which is reversed by ectopic WIP1 expression [31]. *WIP1*, which contains an NF-κB binding site in its promoter region, is a downstream gene target of NF-κB [32]. Interestingly, β-catenin associates with NF-κB and induces the expression of NF-κB target genes [33] such as WIP1.

Thus, our investigation examines the possibility that WIP1 expression is regulated by β-catenin and that WIP1-associated p53-mediated senescence accounts for phenotypes resulting from CDK5RAP2 loss-of-function. Since β-catenin is phosphorylated by GSK3β that earmarks β-catenin for degradation [34], we further examined whether GSK3β controls our presumed β-catenin-mediated WIP1 expression. Using BJ fibroblasts and *Cdk5rap2*^*an/an*^ mice [35] and MEFs, we demonstrate that CDK5RAP2 loss-of-function triggers premature cell senescence. Proliferation defect and senescent phenotypes in CDK5RAP2-depleted BJ cells are recapitulated in *Cdk5rap2*^*an/an*^ MEFs and embryos, manifesting as reduced growth rate and reduced embryonic body weight and size, respectively, as well as increased SA-β-gal staining in both MEFs and embryos. Our findings demonstrate that premature cell senescence due to CDK5RAP2 loss-of-function occurs via elevation of GSK3β activity that causes β-catenin-mediated downregulation of WIP1 and subsequent upregulation of p53 Ser15 phosphorylation.

## Results

### Loss of CDK5RAP2 induces senescence

**Fig. 1:**
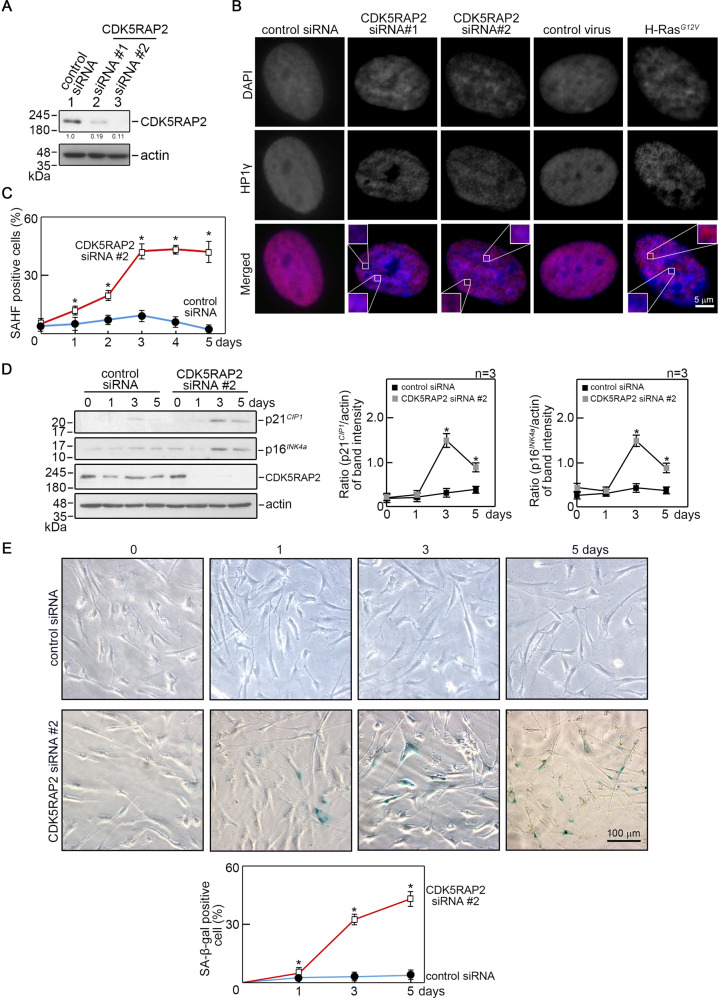
CDK5RAP2 loss triggers SAHF formation and increased SA-β-gal staining. **A** Depletion of CDK5RAP2 in BJ human diploid foreskin fibroblasts. Lysates of cells transfected with CDK5RAP2 siRNA for 3 days were analyzed by SDS-PAGE and immunoblotting for CDK5RAP2 (left panel). Actin blot was used as a loading control. Representative blots are from one of three independent experiments (*n* = 3) showing similar results. The numbers under the CDK5RAP2 bands represent the ratios of the densitometric levels of CDK5RAP2 vs actin, with values from cells transfected with control siRNA normalized to 1.0. Densitometric analysis of blots was performed using NIH Image J 1.61. **B** CDK5RAP2 loss causes the formation of SAHF. BJ cells transfected with CDK5RAP2 siRNA for 3 days were stained with DAPI and HP1γ antibody and subjected to microscopic examination. SAHF formation induced by infecting adenovirus carrying H-RAS^*G12V*^ into BJ cells was used as a positive control. Images shown are of single-cell nuclei from control and CDK5RAP2-depleted cells, and cells infected with adenovirus carrying GFP alone or H-RAS^*G12V*^. Inset images show differences in DAPI and HP1γ staining. **C**, **D** BJ cells transfected with CDK5RAP2 siRNA #2 for 5 days were analyzed for SAHF positive cells (**C**) and by SDS-PAGE and immunoblotting for p21^*CIP1*^ and p16^*INK4a*^ as well as CDK5RAP2 and actin (**D**). The number of SAHF positive cells was assessed in ~200 cells per treatment group in each of 3 independent experiments (*n* = 3). *p < 0.01. In **D**, actin was used as a loading control. Representative blots are from one of three independent experiments (*n* = 3) showing similar results. Ratios of levels of p21^*CIP1*^ and p16^*INK4a*^ vs. actin and standard deviation for the 3 independent sets of experiments (right panels) were calculated as described for CDK5RAP2 vs actin above, with values from cells transfected with CDK5RAP2 siRNA #2 at day 5 normalized to 1.0. *p < 0.02. **E** Cells transfected with CDK5RAP2 siRNA #2 or control siRNA were subjected to SA-β-gal staining at days 1, 3, and 5 post-transfection. Representative images (upper panel) are from one of three independent experiments (*n* = 3) showing similar staining patterns. The number of SA-β-gal positive cells was assessed in ~200 cells per treatment group in each of the 3 independent experiments (*n* = 3). *p < 0.001.

To investigate how CDK5RAP2 loss-of-function causes proliferation defects associated with developmental disorders, we examined the effect of CDK5RAP2 depletion in BJ fibroblasts by siRNA. As shown in Fig. 1, knocking down CDK5RAP2 using two different siRNAs (#1 and #2) triggers the formation of SAHF as demonstrated by DAPI staining, and SAHF colocalization with heterochromatin protein 1γ (HP1γ), a SAHF marker [36] (Fig. 1B). Senescence induced by activated H-RAS^*G12V*^ oncogene [37] was used as a positive control to detect SAHF and HP1γ staining. To explore the suggestion that CDK5RAP2 depletion causes premature cell senescence, cells transfected with CDK5RAP2 siRNA #2 were monitored for the appearance of SAHF-positive cells and the expression of the senescence-associated biomarkers [36], p21^*CIP1,*^ and p16^*INK4a*^, over 5 days post-transfection. As shown in Fig. 1C, the number of SAHF positive cells increases markedly in the first 3 days after transfection with CDK5RAP2 siRNA #2, reaching peak levels on days 3–5, a period when no or only a modest number of SAHF positive cells was observed in cells transfected with control siRNA. The increase in the number of SAHF positive cells upon CDK5RAP2 depletion coincides with increased expression of p21^*CIP1*^ and p16^*INK4a*^ (Fig. 1D). Together with the noticeable staining for SA-β-gal, another marker of cellular senescence [38], in cells transfected with CDK5RAP2 siRNA #2 (Fig. 1E), our findings indicate that loss of CDK5RAP2 induces premature cellular senescence. To substantiate the occurrence of SA-β-gal positive cells following CDK5RAP2 loss, endogenous CDK5RAP2 was depleted in BJ cells using CDK5RAP2 siRNA #2 then exogenous CDK5RAP2 was overexpressed by infection of lentivirus carrying GFP-CDK5RAP2. As shown in Supplementary Fig. 1, exogenous CDK5RAP2 expression reversed the occurrence of SA-β-gal positive cells, which was clearly increased upon endogenous CDK5RAP2 depletion. Together, these findings indicate that CDK5RAP2 loss promotes premature cell senescence.

Increased levels of SAHF, as well as p21CIP1 and p16INK4a, that also inhibit cell cycle Cdks, upon loss of CDK5RAP2, led us to test whether CDK5RAP2 loss affects cell proliferation. Indeed, we found that CDK5RAP2-depleted cells proliferate at a slower rate compared to control cells (Supplementary Fig. 2A). Flow cytometry showed an increased population of cells at G_0_/G_1_, but a reduced population of cells at S in CDK5RAP2-depleted cells compared to control cells (Supplementary Fig. 2B). In addition, a reduced number of Ki-67 positive cells was observed in CDK5RAP2-depleted cells compared to control cells (Supplementary Fig. 2C). Altogether, these findings indicate that CDK5RAP2 loss triggers senescence-associated proliferation arrest.

### Reduced body weight and size of *Cdk5rap2*^*an/an*^ embryos are associated with increased senescence and reduced proliferation in corresponding MEFs

**Fig. 2:**
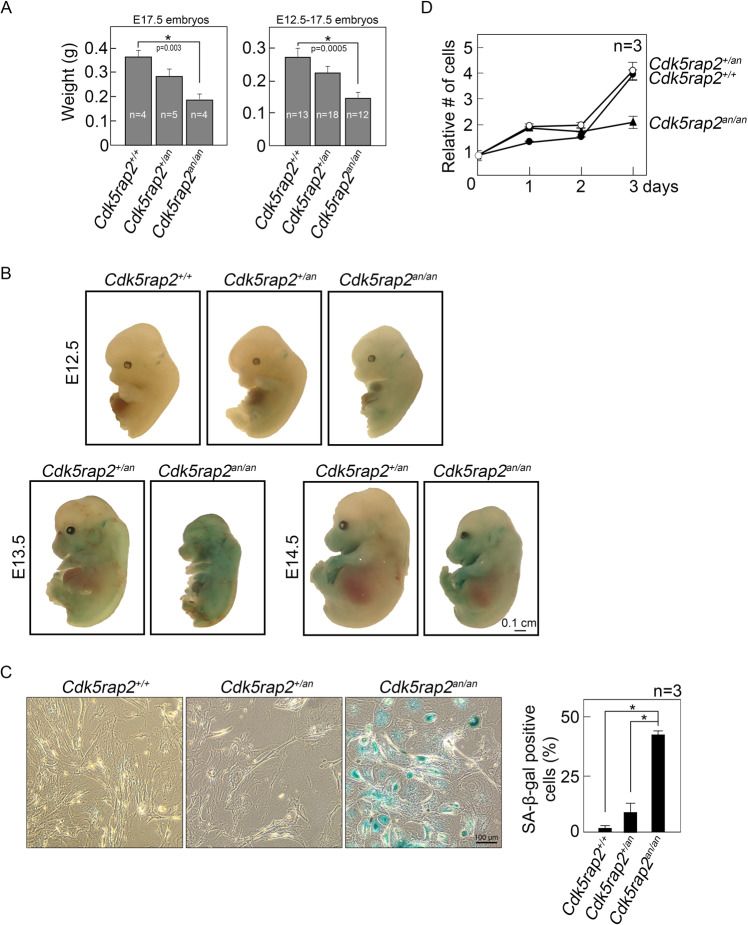
*Cdk5rap2*^*an/an*^ embryos with reduced body weight exhibit elevated SA-β-gal staining compared to *Cdk5rap2*^*+/an*^ and *Cdk5rap2*^*+/+*^ embryos. **A** Average weights of isolated E17.5 (left panel) and E12.5 to E17.5 (right panel). *Cdk5rap2*^*+/+*^, *Cdk5rap2*^*+/an*^, and *Cdk5rap2*^*an/an*^ embryos from different litters are shown. **B** SA-β-gal staining of E12.5-E14.5 *Cdk5rap2*^*an/an*^ and *Cdk5rap2*^*+/an*^ and *Cdk5rap2*^*+/+*^ littermate embryos was performed as described in Materials and Methods. **C** MEFs isolated from *Cdk5rap2*^*+/+*^, *Cdk5rap2*^*+/an,*^ and *Cdk5rap2*^*an/an*^ embryos were subjected to SA-β-gal staining. Representative images (left panel) are from one of three independent experiments (*n* = 3) showing similar staining patterns. The number of SA-β-gal positive cells was assessed in ~200 cells per treatment group in each of the 3 independent experiments (*n* = 3). *p = 0.0002. **D** Growth of MEFs obtained from *Cdk5rap2*^*+/+*^, *Cdk5rap2*^*+/an*^, and *Cdk5rap2*^*an/an*^ embryos were analyzed by cell viability assay.

The availability of Hertwig’s anemia (an) mutant mice (*Cdk5rap2*^*an/an*^) that carry exon 4 inversions in the *Cdk5rap2* gene, allowed us to test whether such *Cdk5rap2*^*an/an*^ mutation affects body weight and size, and whether the senescence-associated phenotypes observed in CDK5RAP2-deficient BJ cells are also exhibited in *Cdk5rap2*^*an/an*^ embryos and MEFs. Since *Cdk5rap2*^*an/an*^ embryos are likely to die in late embryonic stages, we isolated and compared body weights of embryonic day 17.5 (E17.5) *Cdk5rap2*^*+/+*^, *Cdk5rap2*^*+/an,*^ and *Cdk5rap2*^*an/an*^ embryos from pregnant *Cdk5rap2*^*+/an*^ mice. As shown in Fig. 2A, E17.5 *Cdk5rap2*^*an/an*^ embryos weigh less than the *Cdk5rap2*^*+/+*^ and *Cdk5rap2*^*+/an*^ embryos. A similar pattern was noted for average weights of E12.5 to E17.5 *Cdk5rap2*^*+/+*^, *Cdk5rap2*^*+/an,*^ and *Cdk5rap2*^*an/an*^ embryos (Fig. 2A, right panel). We then tested whether *Cdk5rap2*^*an/an*^ embryos with reduced body weight exhibit increased senescence compared with *Cdk5rap2*^*+/an*^ and *Cdk5rap2*^*+/+*^ embryos. To do so, E12.5-E14.5 littermate embryos were subjected to whole-mount SA-β-gal staining. As shown in Fig. 2B, *Cdk5rap2*^*an/an*^ embryos with reduced body weight display increased SA-β-gal staining compared to *Cdk5rap2*^*+/+*^ and *Cdk5rap2*^*+/an*^ embryos. Next, MEFs isolated from E12.5 *Cdk5rap2*^*+/+*^, *Cdk5rap2*^*+/an*^ and *Cdk5rap2*^*an/an*^ embryos were examined for the appearance of SA-β-gal positive cells. Figure 2C (left panel) shows that the number of SA-β-gal positive cells in *Cdk5rap2*^*an/an*^ MEFs is remarkably greater than those in *Cdk5rap2*^*+/+*^ and *Cdk5rap2*^*+/an*^ MEFs. In addition, *Cdk5rap2*^*an/an*^ MEFs exhibit decreased proliferation (Fig. 2D) compared to *Cdk5rap2*^*+/+*^ and *Cdk5rap2*^*+/an*^ MEFs. Thus, the senescence-associated phenotypes that we observed in CDK5RAP2-depleted BJ cells arerecapitulated in *Cdk5rap2*^*an/an*^ embryos and ex vivo MEFs.

### Cell senescence due to loss of CDK5RAP2 is linked to increased p53 Ser15 phosphorylation via WIP1 downregulation

**Fig. 3:**
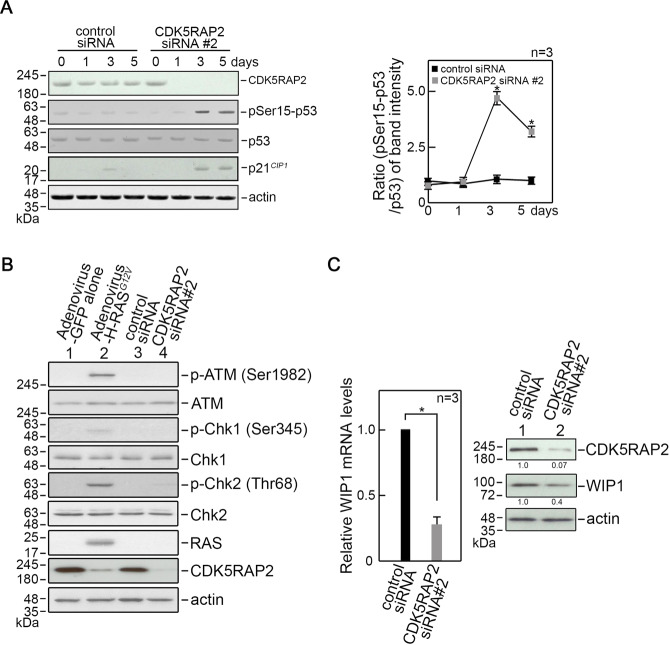
CDK5RAP2 loss triggers cellular senescence through downregulation of WIP1-mediated p53 activation. **A** Lysates of cells transfected with CDK5RAP2 siRNA #2 for 1, 3, and 5 days were analyzed by SDS-PAGE and immunoblotting for CDK5RAP2, phosphoSer15-p53 (pSer15-p53), p53, p21^*CIP1*^*,* and actin (**A**, left panel). Actin blot was used as a loading control. Representative blots are from one of three independent experiments (*n* = 3) showing similar results. Ratios of levels of phosphoSer15-p53 (pSer15-p53) vs. total p53 and standard deviation for the 3 independent sets of experiments (**A**, right panel) were calculated as described for CDK5RAP2 vs actin in Fig. 1, with values from cells transfected with control siRNA at day 0 normalized to 1.0. *p < 0.005. **B** Lysates of cells infected with adenovirus carrying H-RAS^*G12V*^ or transfected with CDK5RAP2 siRNA #2 for 3 days were analyzed by SDS-PAGE and immunoblotting for CDK5RAP2, phosphoSer1982-ATM (pSer1982-ATM), ATM, phosphoSer345-Chk1 (pSer345-Chk1), Chk1, phosphoThr68-Chk2 (pThr68-Chk2), Chk2, H-RAS, and Actin. Actin blot was used as a loading control. **C** CDK5RAP2 loss represses WIP1 mRNA and protein expression. Levels of WIP1 mRNA in cells transfected with CDK5RAP2 siRNA #2 were determined by qRT-PCR using isolated total RNA as template. GAPDH was used for normalization. Relative levels of WIP1 mRNA (left panel) were calculated using the 2^−ΔΔCT^ method. Relative levels from cells transfected with control siRNA were normalized to 1.0. Data represent means ± SD of calculated ratios from three independent experiments (*n* = 3). *p = 0.002. WIP1 protein expression was analyzed by immunoblotting. Actin blot was used as a loading control. Representative blots from one of three independent experiments (*n* = 3) showing similar results are shown. Ratios of levels of WIP1 vs actin for the 3 independent sets of experiments were calculated as described for CDK5RAP2 vs actin in Fig. 1, with values from cells transfected with control siRNA normalized to 1.0. *p < 0.01.

p53 plays a key role in triggering SA-β-gal expression. It is phosphorylated at Ser15 by ATM, Chk1, or Chk2 [22–24]. Upon DNA damage and presence of other stressors that induce cellular senescence [25, 39]. Thus, we examined the phosphorylation status of p53 Ser15 in CDK5RAP2-depleted BJ cells. By immunoblotting, we observed that CDK5RAP2 loss causes a noticeable increase in p53 Ser15 phosphorylation, which coincides with elevated p21^*CIP1*^ level (Fig. 3A). Next, we tested whether senescence due to CDK5RAP2 loss involves ATM, Chk1, and/or Chk2 activation. Phosphorylation of ATM Ser1981, Chk1 Ser345, and Chk2 Thr68 was examined in CDK5RAP2-depleted cells by immunoblotting. Cells infected with H-RAS^*G12V*^ oncogene [37] were used as positive control to detect senescence-associated activation of ATM, Chk1, and Chk2. Interestingly, we did not detect activation of ATM, Chk1 or Chk2 in CDK5RAP2-depleted cells (Fig. 3B), and observed reduced CDK5RAP2 level in cells infected with activated H-RAS^*G12V*^. The mechanism by which the latter happens requires further investigation but is beyond the scope of the current study. Since p53 Ser15 phosphorylation is also regulated by protein phosphatases such as WIP1, a critical senescence regulator [28, 29] that dephosphorylates p53 Ser15, we examined the possibility that CDK5RAP2 loss affects WIP1 expression. By qRT-PCR and immunoblotting, we found that WIP1 phosphatase is downregulated at both the mRNA (Fig. 3C, left panel) and protein (Fig. 3C, right panel) levels in CDK5RAP2-depleted cells. Taken together, our findings indicate that premature cell senescence due to CDK5RAP2 loss may result from p53 phosphorylation at Ser15 due to downregulation of WIP1, and that p53-mediated senescence due to CDK5RAP2 loss is independent of ATM, Chk1, or Chk2 activation.

While we note that p53-associated cell senescence induced by CDK5RAP2 loss is distinct from p53-mediated senescence induced by activated H-RAS^*G12V*^, which is often associated with DDR activation [19, 21–23], we sought to examine the possibility that senescence due to CDK5RAP2 loss involves DDR. To do so, we performed immunofluorescence microscopy to test whether CDK5RAP2 loss in BJ cells triggers γH2AX foci formation (Supplementary Fig. 3). Cells infected with activated H-RAS^*G12V*^ or treated with 4 Gy ionizing radiation (IR), which show 96.7% and 100%, respectively, of cells positive for γH2AX foci served as positive controls. Cells infected with an empty vector or transfected with control siRNA served as negative controls. Examination of CDK5RAP2-depleted cells revealed no γH2AX foci, indicating that p53-associated senescence is linked to WIP1 downregulation in CDK5RAP2-depleted cells does not involve DDR activation.

**Fig. 4:**
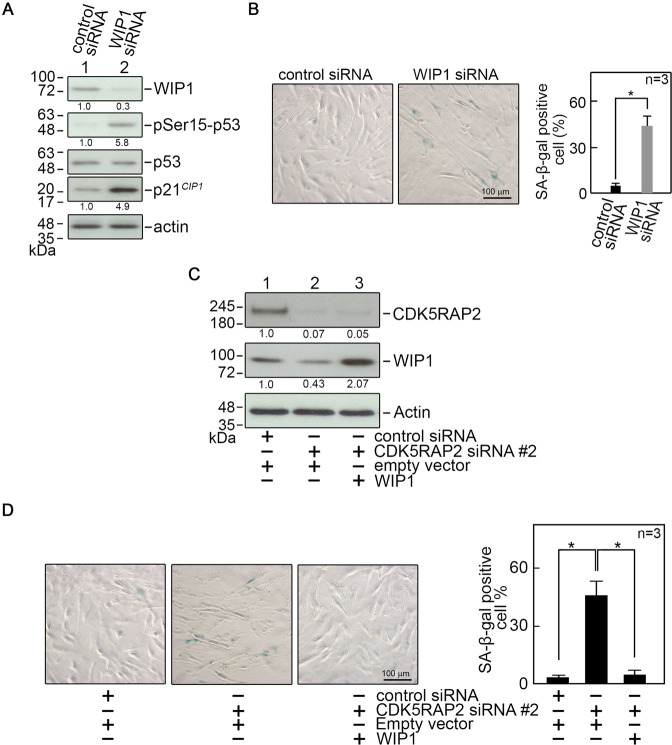
WIP1 loss triggers cellular senescence through p53 activation and ectopic expression of WIP1 reverses the senescent phenotypes observed in cells depleted of CDK5RAP2. **A** BJ cells transfected with WIP1 siRNA were analyzed by SDS-PAGE and immunoblotting for WIP1, pSer15-p53, p53, and p21^*CIP1*^. Actin was used as a loading control. Representative blots are from one of three independent experiments (*n* = 3) showing similar results. The numbers under the WIP1, pSer15-p53, and p21^*CIP1*^ bands represent the ratios of the densitometric levels of WIP1 vs actin, pSer15-p53 vs total p53, and p21^*CIP1*^ vs actin, with values from cells transfected with control siRNA normalized to 1.0. **B** BJ cells transfected with WIP1 siRNA were analyzed by SA-β-gal staining. Representative images of SA-β-gal staining (left panel) are from one of three independent experiments (*n* = 3) showing similar staining patterns. The number of SA-β-gal positive cells (**B**, right panel) was assessed in ~200 cells per treatment group in each of the 3 independent experiments (*n* = 3). *p = 0.00016. **C**, **D** Lysates of BJ cells co-transfected with CDK5RAP2 siRNA #2 and pReceiver-M02 carrying WIP1 were analyzed by SDS-PAGE and immunoblotting for CDK5RAP2 and WIP1 (**C**) and SA-β-gal staining (**D**). Actin was used as loading control. Representative blots are from one of three independent experiments (*n* = 3) showing similar results. The numbers under the CDK5RAP2 and WIP1 bands represent the ratios of the densitometric levels of CDK5RAP2 or WIP1 vs actin, with values from cells co-transfected with control siRNA and empty vector normalized to 1.0. Representative images of SA-β-gal staining in **D** (left panel) are from one of three independent experiments (*n* = 3) showing similar staining patterns. The number of SA-β-gal positive cells (**D**, right panel) was assessed in ~100 cells per treatment group in each of the 3 independent experiments (*n* = 3). *p < 0.001.

To examine the idea that WIP1 loss in CDK5RAP2-depleted cells accounts for p53 Ser15 phosphorylation that leads to cellular senescence, WIP1- depleted BJ cells were analyzed by SDS-PAGE and immunoblotting for WIP1, p53 phophoSer15, total p53, and p21^*CIP1*^ as well as SA-β-gal staining. Consistent with our observations in CDK5RAP2-depleted cells, WIP1-depleted cells show p53 Ser15 phosphorylation, an increased p21^*CIP1*^ level (Fig. 4A), and a number of SA-β-gal positive cells (Fig. 4B). To establish a link between senescence induced by CDK5RAP2 loss and downregulated WIP1 expression, BJ cells were co-transfected with CDK5RAP2 siRNA #2 and pReceiver-M02 vector carrying WIP1. As shown in Fig. 4C, ectopic WIP1 overexpression in CDK5RAP2-depleted cells (Fig. 4C) reverses the appearance of SA-β-gal positive cells induced by CDK5RAP2 loss (Fig. 4D), supporting our view that senescence due to CDK5RAP2 loss is linked to downregulated WIP1 expression.

### CDK5RAP2 regulates WIP1 expression through β-catenin

**Fig. 5:**
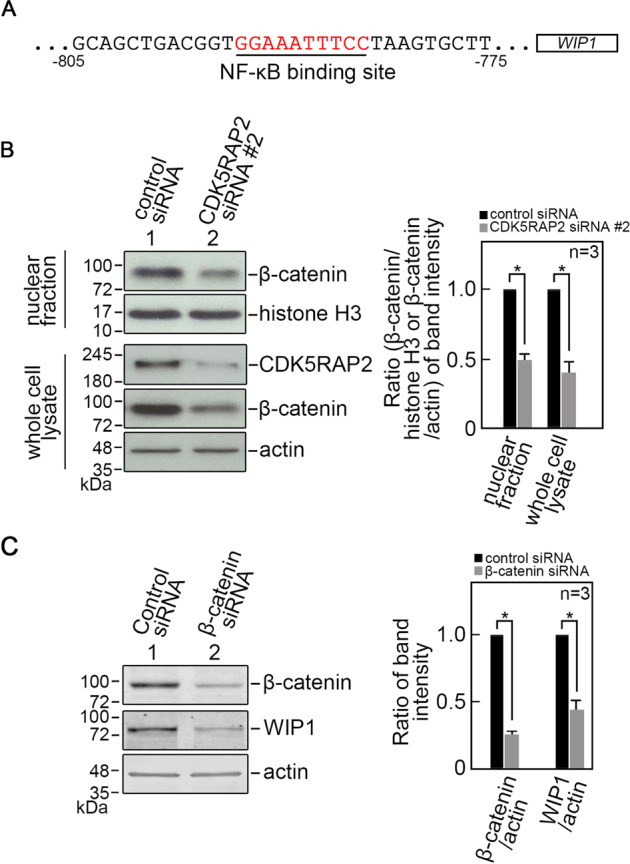
CDK5RAP2 loss reduces β-catenin level. **A** The WIP1 promoter contains an NF-κB binding site [32]. BJ cells transfected with CDK5RAP2 siRNA #2 (**B**), or β-catenin siRNA (**C**) for 3 days were analyzed by SDS-PAGE and immunoblotting. Nuclear fractions and whole-cell lysates in (**B**) were probed for β-catenin and histone H3 (nuclear fraction) or CDK5RAP2 and actin (whole cell lysate), and whole cell lysates in (**C**) were probed for β-catenin, WIP1, and actin. Actin blot was used as a loading control. Representative blots are from one of three independent experiments (*n* = 3) showing similar results. Ratios of levels of β-catenin vs histone H3 (in nuclear fraction) or actin (in whole cell lysate) in (**B**) and β-catenin or WIP1 vs actin in (**C**), right panels, and standard deviation for the 3 independent sets of experiments were calculated as described for CDK5RAP2 vs actin in Fig. 1, with values from cells transfected with control siRNA normalized to 1.0. *p < 0.05 (in **B**). *p < 0.005 (in **C**).

As indicated above, we found that loss of CDK5RAP2 causes downregulation of WIP1 expression at both the mRNA and protein levels. The WIP1 promoter contains a nuclear factor-κB (NF-κB) binding site [32] (Fig. 5A) through which NF-κB positively regulates WIP1 expression [32]; β-catenin associates with NF-κB, affecting the expression of NF-κB target genes [33]. These interactions led us to test whether CDK5RAP2 loss, which reduces WIP1 level, influences β-catenin expression. As shown in Fig. 5B, CDK5RAP2 loss reduces β-catenin level in the nucleus where β-catenin associates with NF-κB. β-catenin depletion downregulates WIP1 (Fig. 5C), suggesting that CDK5RAP2 loss may reduce WIP1 level through β-catenin downregulation.

### CDK5RAP2 interacts with glycogen synthase kinase-3β (GSK3β) and such interaction inhibits GSK3β activity

**Fig. 6:**
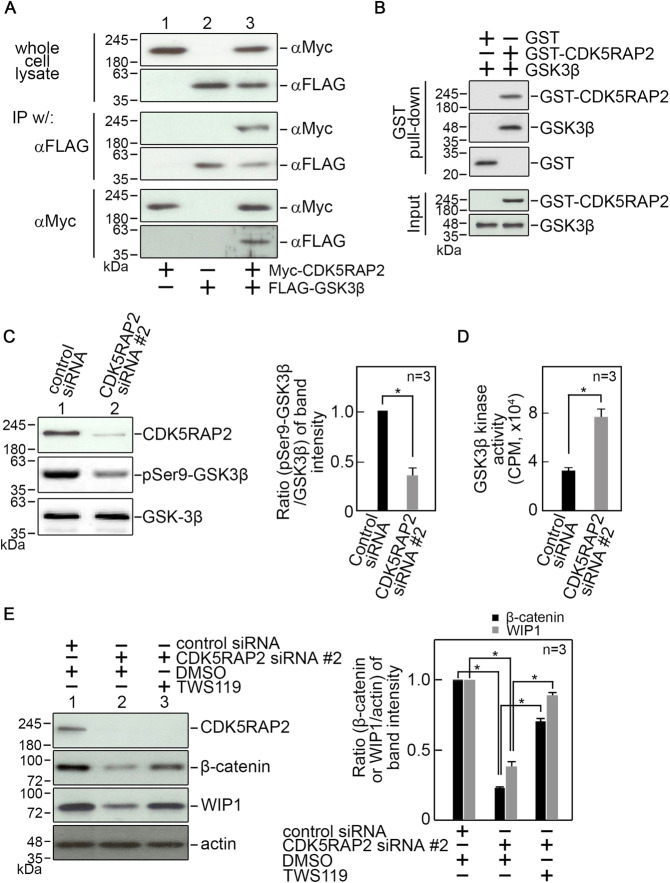
GSK3β interaction with CDK5RAP2 inhibits its kinase activity. **A** Lysates of BJ cells co-transfected with Myc-tagged CDK5RAP2 and FLAG-tagged GSK3β (upper panel) were subjected to immunoprecipitation (IP) using FLAG antibody (middle panel) or Myc antibody (lower panel) then analyzed for CDK5RAP2 and GSK3β co-immunoprecipitation by immunoblotting both IPs with Myc and FLAG antibodies. B. CDK5RAP2 directly interacts with GSK3β. GST-CDK5RAP2 bound to GSH-agarose beads was mixed with GSK3β as described in Materials and Methods. The resulting complexes were washed extensively then analyzed by SDS-PAGE and immunoblotting for GST and GSK3β. **C** Lysates of cells transfected with CDK5RAP2 siRNA #2 for 3 days were analyzed by SDS-PAGE and immunoblotting for CDK5RAP2, phosphoSer9-GSK3β, and GSK3β (left panel). Representative blots are from one of three independent experiments (*n* = 3) showing similar results. Ratios of levels of phosphoSer9-GSK3β vs GSK3β and standard deviation for the 3 independent sets of experiments (right panel) were calculated as described for CDK5RAP2 vs actin in Fig. 1, with values from cells transfected with control siRNA normalized to 1.0. *p = 0.01. **D** Lysates of cells transfected with CDK5RAP2 siRNA #2 for 3 days were subjected to IP using GSK3β antibody. The IPs were then examined for in vitro GSK3β kinase activity as described in Materials and Methods. Data represent means ± SD from three independent experiments (*n* = 3). *p = 0.0027. **E** Inhibition of GSK3β activity with TWS119 (5 μM in DMSO) in cells depleted of CDK5RAP2 restores β-catenin and WIP1 expression (left panel). Representative blots from one of three independent experiments (*n* = 3) showing similar results are shown. Ratios of levels of β-catenin and WIP1 vs actin and standard deviation for the 3 independent sets of experiments (right panel) were calculated as described for CDK5RAP2 vs actin in Fig. 1, with values from cells transfected with control siRNA and treated with DMSO normalized to 1.0. *p < 0.05.

Because GSK3β phosphorylates β-catenin, targeting β-catenin for ubiquitination-mediated degradation by the proteasome pathway [34], we examined whether CDK5RAP2 interacts with GSK3β and affects GSK3β activity. To do so, lysates of BJ cells co-transfected with Myc-tagged CDK5RAP2 and FLAG-tagged GSK3β were subjected to immunoprecipitation using Myc or FLAG antibody; both immunoprecipitates were then analyzed for CDK5RAP2 and GSK3β co-immunoprecipitation. Fig 6A shows reciprocal co-immunoprecipitation of CDK5RAP2 and GSK3β. To determine whether CDK5RAP2 interacts directly with GSK3β, GST-CDK5RAP2 purified from baculovirus-infected Sf9 insect cells (Supplementary Fig. 4) was bound to GSH-agarose beads then mixed with purified GSK3β. Analysis of the GST pulled-down complex by immunoblotting using GST or GSK3β antibody show the presence of both GST-CDK5RAP2 and GSK3β (Fig. 6B), indicating direct interaction between these proteins. We then sought to determine whether CDK5RAP2 interaction with GSK3β affects GSK3β activity. Since GSK3β phosphorylation at Ser9 inhibits its activity [34, 40], we first examined whether CDK5RAP2 loss influences the state of GSK3β Ser9 phosphorylation. As shown in Fig. 6C, CDK5RAP2-depleted cells have reduced GSK3β Ser9 phosphorylation compared to control cells, suggesting that CDK5RAP2 interaction with GSK3β could affect GSK3β activity. Indeed, by in vitro GSK3β kinase assay, we found that the GSK3β immunoprecipitate from CDK5RAP2-depleted cells show greater GSK3β activity compared to control cells (Fig. 6D). To examine a link between increased GSK3β activity and reduced β-catenin and WIP1 levels in CDK5RAP2-depleted cells, we tested whether TWS119, a potent GSK3β inhibitor, restores β-catenin and WIP1 levels in these cells. Immunoblotting shows that TWS119 restores β-catenin and WIP1 levels in CDK5RAP2-depleted cells (Fig. 6E), supporting a link between increased GSK3β activity and reduced β-catenin and WIP1 levels upon loss of CDK5RAP2.

### CDK5RAP2 loss induces senescence through upregulation of GSK3β activity and subsequent downregulation of β-catenin-mediated WIP1 expression

**Fig. 7:**
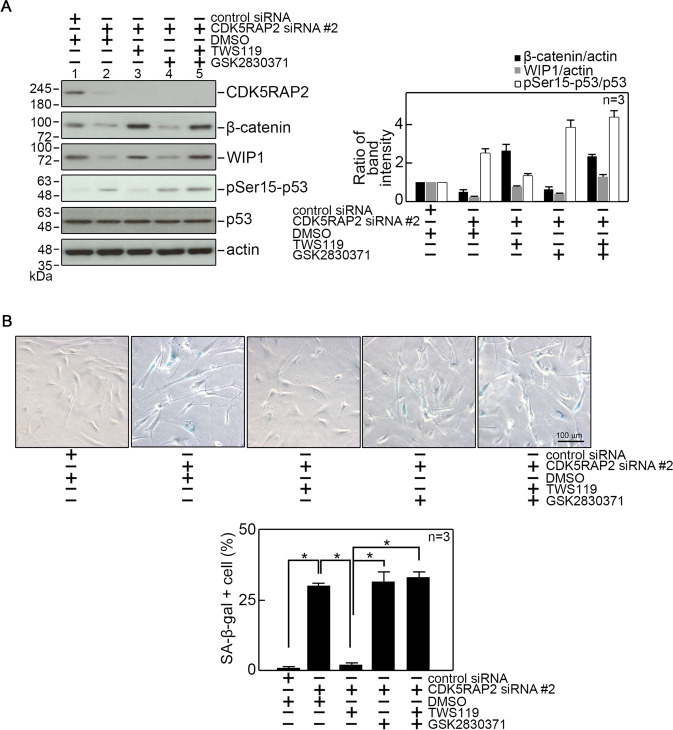
Inhibition of GSK3β in CDK5RAP2-depleted cells inhibits senescence that is induced upon inhibition of WIP1 activity. **A** BJ cells transfected with CDK5RAP2 siRNA #2 were treated with TWS119 (5 μM in DMSO) and/or GSK2830371 (10 μM in DMSO), a potent inhibitor of WIP1 activity. Cell lysates were then analyzed by SDS-PAGE and immunoblotting for CDK5RAP2, β-catenin, WIP1, phosphoSer15-p53, p53, and actin (left panel). Actin blot was used as a loading control. Representative blots from one of three independent experiments (*n* = 3) showing similar results are shown. Ratios of levels of β-catenin and WIP1 vs actin and phosphoSer15-p53 vs. p53, and standard deviation for the 3 independent sets of experiments (right panel) were calculated as described for CDK5RAP2 vs. actin in Fig. 1, with values from cells transfected with control siRNA and treated with DMSO normalized to 1.0. *p < 0.05. **B** Representative images of SA-β-gal staining (upper panel) are from one of three independent experiments (*n* = 3) showing similar staining patterns. The number of SA-β-gal positive cells (lower panel) was assessed in ~100 cells per treatment group in each of the 3 independent experiments (*n* = 3). *p < 0.05.

Next, we examined whether inhibition of GSK3β with TWS119, which restores β-catenin and WIP1 levels in CDK5RAP2-depleted BJ cells, inhibits senescence in these cells. We found that treatment of BJ cells with TWS119, which increases β-catenin and WIP1 levels and reduces p53 Ser15 phosphorylation in CDK5RAP2-depleted cells (Fig. 7A), causes loss of SA-β-gal staining in these cells (Fig. 7B), suggesting that GSK3β inhibition prevents senescence in CDK5RAP2-depleted cells through upregulation of β-catenin and WIP1, and subsequent downregulation of p53 Ser15 phosphorylation. We then examined the effect of GSK2830371, a potent WIP1 inhibitor, in CDK5RAP2-depleted cells treated with TWS119. Fig 7A shows that GSK2830371 has no effect on β-catenin and WIP1 levels in these cells, but causes an increase in p53 Ser15 phosphorylation and thus, as shown in Fig. 7B, an increase in SA-β-gal staining. These findings support our view that GSK3β acts upstream of β-catenin as well as WIP1. Therefore, although GSK3β inhibition prevents senescence in CDK5RAP2-depleted cells by increasing β-catenin and WIP1 levels, which reduces p53 Ser15 phosphorylation, direct inactivation of WIP1 in cells lacking CDK5RAP2 and GSK3β activity results in p53 Ser15 phosphorylation and increased senescence.

**Fig. 8:**
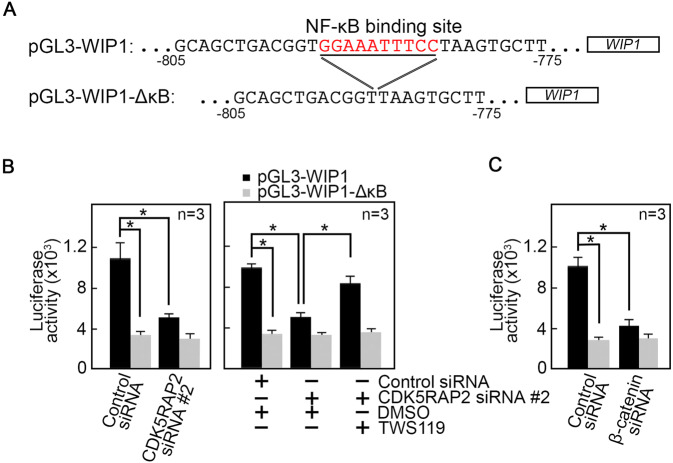
CDK5RAP2 regulates WIP1 promoter activity via β-catenin. **A** Sequence of pGL3 luciferase reporter vector carrying WIP1 or WIP1-ΔκB promoter. **B**, **C** Loss of CDK5RAP2 (**B** left and right panels) or β-catenin (**C**) inhibits WIP1 promoter activity as measured by luciferase reporter activity but inhibition of GSK3β with TWS119 (5 μM in DMSO) in CDK5RAP2-depleted cells restores WIP1 promoter activity (**B**, right panel). Data represent means ± SD from three (*n* = 3) independent experiments. *p < 0.05.

Promoter activity analysis by transfecting a luciferase reporter vector carrying wt WIP1 promoter (pGL3-WIP1, Fig. 8A) into cells depleted of CDK5RAP2 (Fig. 8B) or β-catenin (Fig. 8C) shows reduced luciferase activity in these cells compared to control cells (Fig. 8B, C). Conversely, transfection with WIP1 lacking its NF-κB binding site (pGL3-WIP1-ΔκB; Fig. 8A) has no effect on luciferase activity in CDK5RAP2- or β-catenin-depleted cells. Treatment with TWS119 restored luciferase activity in CDK5RAP2-depleted cells transfected with pGL3-WIP1 (Fig. 8B, right panel), further suggesting that senescence due to CDK5RAP2 loss is controlled by GSK3β through regulation of the WIP1 promoter via β-catenin.

## Discussion

In this study, we provide evidence that CDK5RAP2 loss induces premature cell senescence through upregulation of GSK3β activity, which causes downregulation of β-catenin-modulated WIP1 level and subsequent maintenance of p53 activation through p53 Ser15 phosphorylation. These were demonstrated using BJ human fibroblasts as well as *Cdk5rap2*^*an/an*^ mouse embryos and ex vivo MEF cells. In BJ cells, we eliminated the possibility of CDK5RAP2 siRNA off-target effects by using two different siRNAs (#1 and #2) that both depleted CDK5RAP2 and induced similar senescent phenotypes that were not observed in cells transfected with control siRNA. For subsequent experiments, CDK5RAP2 siRNA #2, which causes greater depletion of CDK5RAP2 was used. Premature BJ cell senescence due to CDK5RAP2 loss was verified by an increase in number of SAHF positive cells, upregulation of the senescence-associated genes, p21^*CIP1*^ and p16^*INK4a*^ [41], and increased SA-β-gal staining. These senescent phenotypes observed in CDK5RAP2-depleted BJ cells are recapitulated in ex vivo MEFs isolated from *Cdk5rap2*^*an/an*^ embryos, and consistent with a previous report that MEFs isolated from mice carrying a frameshift loss-of-function mutation (R1334SfsX5) [6, 42] in *Cdk5rap2* show growth arrest between passages 8 and 10 and stop growing to confluence after then [43]. However, the latter finding was not further investigated. Here, we demonstrate reduced proliferation in CDK5RAP2-depleted BJ cells by increased population of G_0_/G_1_ cells and reduced population of S phase cells. In *Cdk5rap2*^*an/an*^, proliferation defect is manifested by reduced body weights in E17.5 and combined E12.5, E14.5, E15.5, and E17.5 embryos compared to *Cdk5rap2*^*+/+*^ and *Cdk5rap2*^*+/an*^. Increased senescence in E12.5-E14.5 *Cdk5rap2*^*an/an*^ compared with *Cdk5rap2*^*+/+*^ and *Cdk5rap2*^*+/an*^ littermates was further observed in whole-mount embryos stained with β-galactosidase.

Analysis of senescence upon loss of CDK5RAP2 in BJ cells revealed an increase in p53 Ser15 phosphorylation, an early event that leads to senescence [25, 39]. Interestingly, increased p53 Ser15 phosphorylation in senescent CDK5RAP2-depleted cells is not due to activation of the p53 kinases, ATM, Chk1, or Chk2 [24], but instead is due to downregulation of WIP1 phosphatase expression. This concurs with previous reports that link cell senescence to WIP1-mediated p53 dephosphorylation at Ser15 [28, 29]. While p53 phosphorylation at Ser15 was found to inhibit its interaction with the E3 ubiquitin ligase, Mdm2, preventing degradation and stabilizing p53 [24], we did not observe increased p53 level in CDK5RAP2-depleted cells. Since p53 Ser15 phosphorylation is required for p53 activation [25, 26], WIP1 downregulation and subsequent p53 activation in CDK5RAP2-depleted cells likely account for senescence in these cells. Indeed, elevated level of p21^*CIP1*^, a major transcriptional target of p53 [27] that serves as a senescence biomarker [36], was observed upon CDK5RAP2 loss. We note, however, that absence of γH2AX foci in CDK5RAP2-depleted cells indicates that p53-associated senescence that is linked to WIP1 downregulation in these cells is independent of DDR activation. This is consistent with the lack of DDR-associated activation of ATM, Chk1 or Chk2 in CDK5RAP2-depleted cells.

Our finding that knocking down WIP1 in CDK5RAP2-depleted cells causes increased p53 Ser15 phosphorylation and number of SA-β-gal positive cells, while ectopic expression of WIP1 in these cells reverses such phenotypes establishes a link between cellular senescence due to CDK5RAP2 loss and downregulation of WIP1. This is in accord with previous findings that *Wip1*^−/−^ mice exhibit aging phenotypes in their hematopoietic stem cells, including impaired repopulating activity [29], and *Wip1*^−/−^ MEFs [29] and mesenchymal stem cells [30] exhibit premature senescence. In addition, reduced WIP1 level in primary chondrocytes triggers the onset of senescence and loss of chondrocyte phenotype, while overexpression of WIP1 retains their proliferative capacity and delays the onset of senescence [31].

Our observation that CDK5RAP2 loss downregulates WIP1 mRNA and protein levels together with the fact that the WIP1 promoter contains an NF-κB binding site through which NF-κB positively regulates WIP1 expression [32], and β-catenin associates with NF-κB to induce expression of NF-κB target genes [34] led to our view that CDK5RAP2 loss reduces WIP1 level through β-catenin downregulation. Our notion that β-catenin level is regulated by GSK3β activity is based on the fact that GSK3β phosphorylation of β-catenin targets β-catenin for ubiquitination-mediated degradation [34], inhibiting β-catenin translocation into the nucleus [44]. Our finding that CDK5RAP2 interacts with GSK3β and such interaction inhibits GSK3β activity explains how CDK5RAP2 loss downregulates β-catenin level. The Harmonizome database [45], and previous immunoprecipitation and mass spectrometry analyses [9] support our finding that CDK5RAP2 binds to GSK3β. Additional evidence that reflects CDK5RAP2-GSK3β interaction is their colocalization in the centrosome, which duplicates in synchrony with the cell cycle. Indeed, although GSK3β, which promotes mitotic progression, is mainly distributed throughout the cytoplasm, a subpopulation localizes in the centrosome [46]. Recently, phosphorylated GSK3β was mapped to the centrosome [47], where CDK5RAP2 exists [48, 49] and regulates centriole engagement and cohesion during the cell cycle [43]. It remains to be determined whether disturbance of these CDK5RAP2 functions contributes to senescence upon CDK5RAP2 loss. Nonetheless, since AKT (protein kinase B), which regulates centrosome composition and integrity during mitosis [50], phosphorylates GSK3β at Ser9 [51, 52], it is possible that CDK5RAP2 binding to centrosome-recruited GSK3β facilitates the inhibitory phosphorylation of GSK3β Ser9 by AKT. Potentially, loss of CDK5RAP2 allows the release of unphosphorylated and active GSK3β from the centrosome, causing GSK3β phosphorylation of β-catenin, which is then ubiquitinated and degraded. Reduced β-catenin level leads to reduced NF-κB-mediated WIP1 phosphatase expression, which results in sustained p53 Ser15 phosphorylation that promotes p53-mediated p21^*CIP1*^ expression, and ultimately induces cell senescence. Our finding that GSK3β inhibition restores β-catenin and WIP1 levels in CDK5RAP2-deficient cells establishes a link between increased GSK3β activity and decreased β-catenin and WIP1 levels in these cells. The fact that luciferase activity is reduced in WIP1 promoter-transfected cells lacking CDK5RAP2 or β-catenin, and GSK3β inhibition restores luciferase activity in *Wip1* promoter-transfected cells depleted of CDK5RAP2, further suggest that senescence caused by CDK5RAP2 loss is controlled by GSK3β through β-catenin regulation of the WIP1 promoter.

In summary, our studies reveal that CDK5RAP2 loss triggers premature cell senescence via β-catenin-mediated downregulation of WIP1. Such senescence is associated with proliferation errors in both human fibroblasts and *Cdk5rap2*^*an/an*^ MEFs and embryos. As CDK5RAP2 loss-of-function has been linked to developmental defects, it is possible that premature cell senescence contributes to these defects.

## Materials and methods

### Materials

hTERT-immortalized BJ human foreskin fibroblast cells and adenovirus carrying H-Ras^*G12V*^ and GFP alone were obtained from Drs. Tara Beattie and Karl Riabowol, respectively, at the University of Calgary. HEK293 cells were from ATCC (Manassas, VA, USA). The CDK5RAP2 antibody (A300-554A) was from Bethyl Laboratories (Montgomery, TX, USA). p21^*CIP1*^ (sc-271610), p16^*INK4a*^ (sc-468), p53 (sc-393031), Ras (sc-35), histone H3 (sc-517576) and actin (sc-8432) antibodies, and protein A/G PLUS-agarose (sc-2003) were from Santa Cruz Biotech (Manassas, VA, USA). phosphoSer15-p53 (#9284), GSK3β (#12456), phosphoSer9-GSK3β (#5558), ATM (#92356), phosphoSer1981-ATM (#13050), Chk1 (#2360), phosphoSer345-Chk1 (#2348), Chk2 (#6334), phosphoThr68-Chk2 (#2197), β-catenin (#8480), GST (#2624) and Ki-67 (#9129) antibodies were from Cell Signaling (Danvers, MA, USA). WIP1 antibody (ab236515) was from Abcam (Cambridge, MA, USA). The HRP-conjugated anti-rabbit (#7074) and anti-mouse (#7076) secondary antibodies were from Cell Signaling (Danvers, MA, USA). Primers and siRNAs were synthesized at the University of Calgary Core DNA services or GenePharma, Shanghai, China. Goat anti-mouse or anti-rabbit secondary antibodies conjugated with Alexa-488 or -594 were from Invitrogen (Carlsbad, CA, USA). Myc-tagged CDK5RAP2 and Flag-tagged GSK3β expression vectors, pReceiver-M02 carrying WIP1, and lentivirus carrying GFP-CDK5RAP2 (EX-E1869-Lv182) were from GeneCopoeia (Rockville, USA). GSK3β protein (14-306) was from Upstate Biotech. (Lake Placid, NY, USA). The GSK3β inhibitor, TWS119 (S1590), and the WIP1 inhibitor, GSK2830371, were from Selleck Chemicals (Houston, TX, USA).

### Animals

The *Cdk5rap2*^*+/an*^ breeding pairs (C57 BL/6 J) were purchased from JAX laboratory (Bar Harbor, ME, USA) and fed with commercial mice chow, provided water ad libitum and kept on a 12:12 hr light-dark cycle. All *Cdk5rap2*^*+/+*^, *Cdk5rap2*^*+/an*^ and *Cdk5rap2*^*an/an*^ embryos isolated from pregnant *Cdk5rap2*^*+/an*^ female mice (2–6 month old) were included for body weight analysis. No randomization and blinding were performed. All animal studies conformed to regulatory standards and were approved by the University of Calgary Health Sciences Animal Care Committee.

### Genotyping

Genomic DNAs were extracted from mice using the Extracta DNA prep kit (Quantabio, Edmonton, AB. Canada). Tissue samples were added to 50–100 µl of extraction buffer, incubated at 95 °C for 30 min, and stabilized with an equal volume of stabilization buffer. DNA sample (1 µl) was added to 10 µl of Quantabio’s AccuStart II PCR SuperMix containing 100 nM of the appropriate DNA primers. The forward primers used were: GAAACCAGGGTGACA GGTACA (wt *Cdk5rap2*) and AGATGTCATGTCTAAAGCAATCACT (an *Cdk5rap2*). The reverse primer used was the same for the wt and an reactions: CCTTTGTCTTTCTGCCCTGA. Based on the expected size of each fragment, the wt *Cdk5rap2* allele corresponds to a 581 bp band whereas the mutant an *Cdk5rap2* allele corresponds to a 500 bp band. The reaction mixtures were input into a Bio-Rad thermocycler using PCR settings recommended by the Jackson Laboratory.

### Cell culture

BJ cells were cultured in Eagle’s minimal essential medium (EMEM, Lonza) containing 10% fetal bovine serum (FBS, GIBCO), and 50 U/ml penicillin and 50 mg/ml streptomycin (Invitrogen, Carlsbad). HEK293 human embryonic kidney cells were cultured in Dulbecco’s modified Eagle medium (DMEM, Invitrogen), containing 10% FBS, 50 U/ml penicillin and 50 mg/ml streptomycin. Cells were maintained at 37 °C in a 5% CO_2_ humidified incubator. After recovering from cryopreservation, BJ cells were used for up to 10 additional population doublings to maintains many characteristics of normal primary cells. Cells were tested for mycoplasma contamination.

### Isolation of primary MEFs

Primary MEFs were isolated from E12.5 *Cdk5rap2*^*+/+*^, *Cdk5rap2*^*+/an*^ and *Cdk5rap2*^*an/an*^ embryos as described previously [53]. Briefly, embryos were washed with 1x PBS, decapitated and eviscerated then washed again with PBS. Embryos were minced using sterile forceps and placed in 3–5 ml of 0.05% trypsin-EDTA, pipetted up and down to get cells into suspension and incubated at 37 °C for 5 min. Cell suspensions were transferred to tubes containing MEF medium (DMEM-high glucose supplemented with 10% FBS, 50 U/ml penicillin and 50 mg/ml streptomycin (Invitrogen, Carlsbad), and 2 mM GlutaMAX) then centrifuged at 1000 rpm for 5 min. Cell pellets were resuspended in fresh media and plated in 10 cm cell culture dishes. Primary MEFs were cultured in DMEM supplemented with 10% FBS and 50 U/ml penicillin and 50 mg/ml streptomycin (Invitrogen, Carlsbad) under hypoxic condition (5% O_2_ and 5% CO_2_ incubator). All experiments were performed in passage P2-P7 MEFs.

### Plasmid/siRNA transfection and adenovirus infection

Cells cultured ~18 h and at about 60% confluency were transfected using Lipofectamine 2000 (Invitrogen) in serum-free medium, which was replaced with complete medium 5 h post-transfection. Cells were harvested at different time points as indicated. siRNA target sequences are: control, CGUACGCGGAAUACUUCGAUU; CDK5RAP2 #1, GGACGUGUUGCUUCAGAAAUU; CDK5RAP2 #2, GAGUCAGCCUUCUGCUAAAUU; WIP1, CCAAUGAAGAUGAGUUAUAUU; GSK3β, AGGAGACCACGACCUGUUAAUU; β-catenin, CTCGGGATGTTCACAACCGAA. Adenovirus infection was carried out at a MOI of 50–100. Media was then replaced with EMEM containing 10% FBS 24 h post infection and cells were incubated until the indicated time. We have previously tested and established the specificity of CDK5RAP2 siRNA #1 and #2 effects in BJ cells [54].

### RNA extraction and real-time qRT-PCR

Total RNA was extracted using TRIzol reagent (Invitrogen, Carlsbad, CA, USA) according to the manufacturer’s protocol, and transcribed into cDNA using high-capacity cDNA reverse transcription kit (Thermo Fisher, Waltham, MA). Real-time qRT-PCR was performed using power SYBR® green PCR master mix (Thermo Fisher, Waltham, MA), and an Applied Biosystems 7500 real-time PCR machine using a standard protocol. The PCR conditions were 35 cycles at 94 °C for 20 s, 60 °C for 20 s and 72 °C for 35 s. The primer sets used were: WIP1-F, GGGAGTGATGGACTTTGGAA; WIP1-R, CAAGATTGTCCATGCTCACC; GAPDH-F, GGAGCGAGATCCCTCCAAAAT; GAPDH-R, GGCTGTTGTCATACTTCTCATGG. GAPDH was used for normalization.

### Western blot analysis

Cell lysates (50 μg) were resolved by SDS-PAGE, transferred to nitrocellulose membrane, and immunoblotted for CDK5RAP2, p21^*CIP1*^, p16^*INK4a*^, p53, phospho-p53, Ras, histone, GSK3β, phosphoSer9-GSK3β, ATM, phosphoSer1981-ATM, Chk1, phosphoSer345-Chk1, Chk2, phosphoThr68-Chk2, β-catenin, WIP1 and actin. Following incubation with HRP-conjugated anti-rabbit or anti-mouse secondary antibody, immunoreactive bands were detected using the ECL reagent (GE Healthcare, Little Chalfont, Buckinghamshire, UK). Western blot images were obtained using the ChemiDoc™ Imager (Bio-Rad) set at optimal exposure. No enhancements were performed.

### Immunofluorescence microscopy

Cells transfected with CDK5RAP2 or control siRNA on coverslips were fixed with 4% paraformaldehyde/PBS for 10 min, permeabilized using 0.1% Triton X-100/PBS for 10 min, then blocked in 2% BSA/PBS for 1 h at room temperature. Coverslips incubated with the indicated primary antibody for 1 h followed by 20 min incubation with secondary antibodies were washed with 1x PBS, counterstained with DAPI, and mounted on glass slides using ProLong™ Diamond Antifade Mountant (P36961, Invitrogen, Carlsbad, CA, USA). Images were captured using a Zeiss Axiovert 200 microscope. BJ cells transfected with CDK5RAP2 siRNA #2 were analyzed for Ki-67 positive cells 3 days post-transfection.

### Senescence-associated β-galactosidase staining

Cells were fixed using 3% paraformaldehyde in PBS (pH 6.0) and stained with 1 mg/ml 5-bromo-4-chloro-indolyl-β-D-galactopyranoside (X-gal) solution containing 5 mM potassium ferrocyanide, 5 mM potassium ferricyanide, 150 mM NaCl, and 2 mM MgCl_2_ in PBS (pH 6.0) for 16–20 h at 37 °C. For whole-mount SA-β-gal staining, E13.5 embryo littermates from *Cdk5rap2*^*+/an*^ heterozygous crosses were isolated in ice-cold PBS. Tails were cut and used for genotyping as described above. Embryos were then fixed in 4% paraformaldehyde in PBS (pH 7.0) overnight at 4 °C, stained with 1 mg/ml 5-bromo-4-chloro-indolyl-β-D-galactopyranoside (X-gal) in 0.2 M citric acid/sodium phosphate buffer (pH 6.0), containing 5 mM potassium ferrocyanide, 5 mM potassium ferricyanide, 150 mM NaCl and 2 mM MgCl_2_, for 6 h at 37 °C. Images were taken under a dissecting microscope. Representative images are from one of three independent experiments (*n* = 3) showing similar staining patterns.

### Cell viability and cell cycle analyses

BJ cells transfected with CDK5RAP2 siRNA #2 or control siRNA were seeded into 96-well plates at 5000 cells per well 24 h post transfection, then analyzed for cell viability at days 0, 2, and 4 using Cell Counting Kit-8 (CCK-8, Dojindo). For analysis of cell cycle distribution, cells transfected with CDK5RAP2 siRNA #2 or control siRNA were fixed using cold 2% formaldehyde and stained with 7-AAD (5 μg/μl) then subjected to flow cytometry.

### Immunoprecipitation

HEK293 cells transfected with Myc-tagged CDK5RAP2 or Flag-tagged GSK3β or both for 48 h were lysed in ice-cold lysis buffer containing 50 mM Tris/pH 8.0, 150 mM NaCl, 1% NP-40, 10 mM EDTA, 5% glycerol, 1 mM phenylmethylsulfonylflouride (PMSF), 10 µg/ml aprotinin, and 10 µg/ml leupeptin. Lysates were then clarified by centrifuged at 13,000 rpm for 25 min at 4 °C. For immunoprecipitation under denaturing condition, 1% SDS was added to the lysis buffer. Lysates were pre-cleared by adding normal mouse or rabbit IgG + sepharose A/G beads and incubating at 4 °C for 2 h followed by centrifugation at 14,000 rpm for 10 min. One-tenth of the lysates from each sample was retained and used to assess input or total cell lysate in western blots. Pre-cleared cell lysates were subjected to immunoprecipitation using Myc or Flag antibody conjugated to protein A/G agarose beads. After incubating the mixture overnight at 4 °C, immunoprecipitates were washed with lysis buffer three times at 4 °C.

### Measurement of GSK3β activity

GSK3β activity was measured using the GSK-3β activity assay kit (Sigma, ON, Canada) following the manufacturer’s protocol. Briefly, cells transfected with CDK5RAP2 or control siRNA were lysed in ice-cold lysis buffer containing 50 mM Tris/pH 8.0, 150 mM NaCl, 1% NP-40, 10 mM EDTA, 5% glycerol, 1 mM phenylmethylsulfonylflouride (PMSF), 10 µg/ml aprotinin and 10 µg/ml leupeptin. Cell lysates (300 µg) were incubated with 2 µl of anti-GSK-3β and 30 µl EZview Red protein-G affinity gel beads at 4 °C for 3 h. The beads were recovered by centrifugation at 8000 × g for 30 sec and washing with 500 µl of ice-cold lysis buffer. The immunoprecipitates were mixed with 20 µl of reaction mixture containing 25 μCi γ^32^P-ATP and 5 µl of GSK-3β substrate solution, incubated at 37 °C for 30 m, then spotted onto P81 phosphate cellulose membranes. Membranes were washed 4 times with 0.5% phosphoric acid and once with acetone. Counting of incorporated radioactivity was performed using a Beckman scintillation counter.

### Expression and purification of GST- CDK5RAP2

GST-CDK5RAP2 was cloned into pFastBac vectors from which baculovirus was generated according to the Bac-to-Bac® baculovirus protein expression system (Thermo Fisher, Waltham, MA). Sf9 insect cells were infected with P2 baculovirus carrying GST-CDK5RAP2 for 24 h. GST-CDK5RAP2 was purified by affinity chromatography using a glutathione (GSH)-conjugated agarose column (Sigma-Aldrich, St. Louis, MO). Sf9 cell lysates expressing GST-CDK5RAP2 were incubated with GSH-agarose for 1 h at 4 ˚C and washed with 1x PBS containing 1% Triton X-100. Bound proteins were eluted with 10 mM reduced GSH in 50 mM Tris elution buffer (pH 8).

### GST pull-down assay

GST-CDK5RAP2 (1 μmole) bound to glutathione-agarose beads was mixed with GSK3β (2 μg) at 4 °C for 2 h. The pulled-down complex was washed 4 times with ice-cold GST lysis buffer (20 mM Tris-HCl (pH 8.0), 200 mM NaCl, 1 mM EDTA (pH 8.0), 0.5% NP-40, 2 µg/µl aprotinin, 1 µg/µl leupeptin, 0.7 µg/ml pepstatin and 25 µg/ml PMSF) by centrifugation at 2500 rpm for 10 min and analyzed by SDS-PAGE and immunoblotting for GST and GSK3β.

### Generation of promoter constructs of wt WIP1 (pGL3-WIP1) and WIP1 with NF-κB binding site deletion (pGL3-WIP1-ΔκB)

Using genomic DNAs isolated from HEK293 cells as a template, the WIP1 promoter region was amplified by PCR using the primer set: ACATTTTCTTGAGCTGATTTTGCTT (WIP1-F1) and TCGGAGAAGACGCTCACTCC (WIP1-R1). The promoter region for WIP1-ΔκB was generated by PCR using two different sets of primers: ACATTTTCTTGAGCTGATTTTGCTT (WIP1-F1) and GTTTAAAAAGCACtta accgtcagct (WIP1-R2), and ACCGAGACTGTGCagctgacggttaaGTGCTT (WIP1-F2) and TCGGAGAAGACGC TCACTCC (WIP1-R1), with overlapping fragments (lowercase letters) in WIP1-R2 and WIP1-F2. Two PCR products were annealed and used as templates for subsequent 8 fusion PCR cycles [55]. PCR products were then purified using the GeneJET PCR Purification Kit (Thermo Fisher, Waltham, MA) and used as templates for PCR amplification using the WIP1-F1 and WIP1-R2 primer set. Generated WIP1 and WIP1-ΔκB promoter PCR products were cloned into pGL3-basic luciferase reporter vector (Promega, Madison, WI, USA) using XhoI and BglII restriction enzyme sites and designated as pGL3-WIP1 and pGL3-WIP1-ΔκB, respectively. Successful cloning was confirmed by DNA sequencing.

### Luciferase assay

To measure luciferase activity, HEK293 cells were co-transfected with the indicated siRNA, pGL3-WIP1 or pGL3-WIP1-ΔκB and pTK-Renilla luciferase vector using lipofectamine 2000 (Invitrogen, Carlsbad, CA, USA). Cell extracts were prepared 48 h after transfection and luciferase activity was measured using the Dual-Luciferase Reporter assay system (Promega, Madison, WI, USA). Renilla luciferase served as an internal control for normalization.

### Statistical analysis

Student’s t-test (unpaired, two-sided) or one or two-way analysis of variance (ANOVA) was used. Significance was set at *p* < 0.05.

## Supplementary information

Supplementary figure legends

Supplementary Figure 1

Supplementary Figure 2

Supplementary Figure 3

Supplementary Figure 4

Reproduction checklist
