# Supplementary material for: CDK5RAP2 loss-of-function causes premature cell senescence via the GSK3β/β-catenin-WIP1 pathway
Source: Cell Death Dis. 2021 Dec 20;13(1):9. doi: 10.1038/s41419-021-04457-2 (PMC8688469; doi:10.1038/s41419-021-04457-2)
Supplement: Supplementary file 1 — Supplementary figure legends [file 41419_2021_4457_MOESM1_ESM.docx]

# Supplementary Figure Legend

**Supplementary Figure 1. Expression of exogenous CDK5RAP2 in cells depleted of CDK5RAP2 reversed cell senescence.** Lysates of BJ cells transfected with CDK5RAP2 siRNA #2 and infected with lentivirus carrying CDK5RAP2 for 3 days were analyzed by SDS-PAGE and immunoblotting for CDK5RAP2 and actin (A) and SA-β-gal staining (B). Actin blot in A was used as loading control. Representative blots are from one of three independent experiments (n=3) showing similar results. Representative images of SA-β-gal staining (B, upper panel) are from one of three independent experiments (n=3) showing similar staining patterns. The number of SA-β-gal positive cells (B, lower panel) was assessed in at least 100 cells per treatment group in each of the 3 independent experiments (n=3). *p<0.001.

**Supplementary Figure 2. CDK5RAP2 loss causes decreased cell proliferation.** BJ cells transfected with CDK5RAP2 siRNA #2 were subjected to (A) cell viability assay, (B) cell cycle distribution analysis by flow cytometry, and (C) measurement of Ki-67 positive cells as described in Materials and Methods. BJ cells have a small population of G_2_/M cells as they are slow-growing cells. Data represent means ± SD from three separate experiments (n=3). *p<0.01 (in A and B). The percentage (%) of Ki-67 positive cells (C, right panel) were assessed in ~200 cells per treatment group in each of the 3 independent experiments (n=3). *p=0.001.

**Supplementary Figure 3. CDK5RAP2-depleted cells have no detectable γH2AX foci.** BJ cells transfected with CDK5RAP2 siRNA #2 for 3 days were stained with DAPI and γH2AX antibody. Cells infected with adenovirus carrying H-RAS*^G12V^* and cells treated with 4 Gy IR were used as positive controls for γH2AX detection. Merged images of γH2AX and DAPI staining are shown. Representative images are from one of three independent experiments showing similar staining patterns. The indicated percentage (%) of γH2AX positive cells was assessed in ~200 cells per treatment group in each of the 3 independent experiments (n=3).

**Supplementary Figure 4. Purification of GST-CDK5RAP2.** Lysate of Sf9 insect cells expressing GST-tagged CDK5RAP2 was subjected to affinity purification using a glutathione-agarose column. After collecting the flow-through, the column was washed until no more protein was detected in the wash. Bound proteins were eluted from the column with elution buffer containing 10 mM reduced glutathione. Two elution fractions (E1 and E2) were collected and subjected to SDS-PAGE and immunoblotting for CDK5RAP2 (upper panel). TCE staining (lower panel) was performed to assess protein loading and GST-CDK5RAP2 purification.
