# Supplementary figures and images for: CDK5RAP2 loss-of-function causes premature cell senescence via the GSK3β/β-catenin-WIP1 pathway
Source: Cell Death Dis. 2021 Dec 20;13(1):9. doi: 10.1038/s41419-021-04457-2 (PMC8688469; doi:10.1038/s41419-021-04457-2)

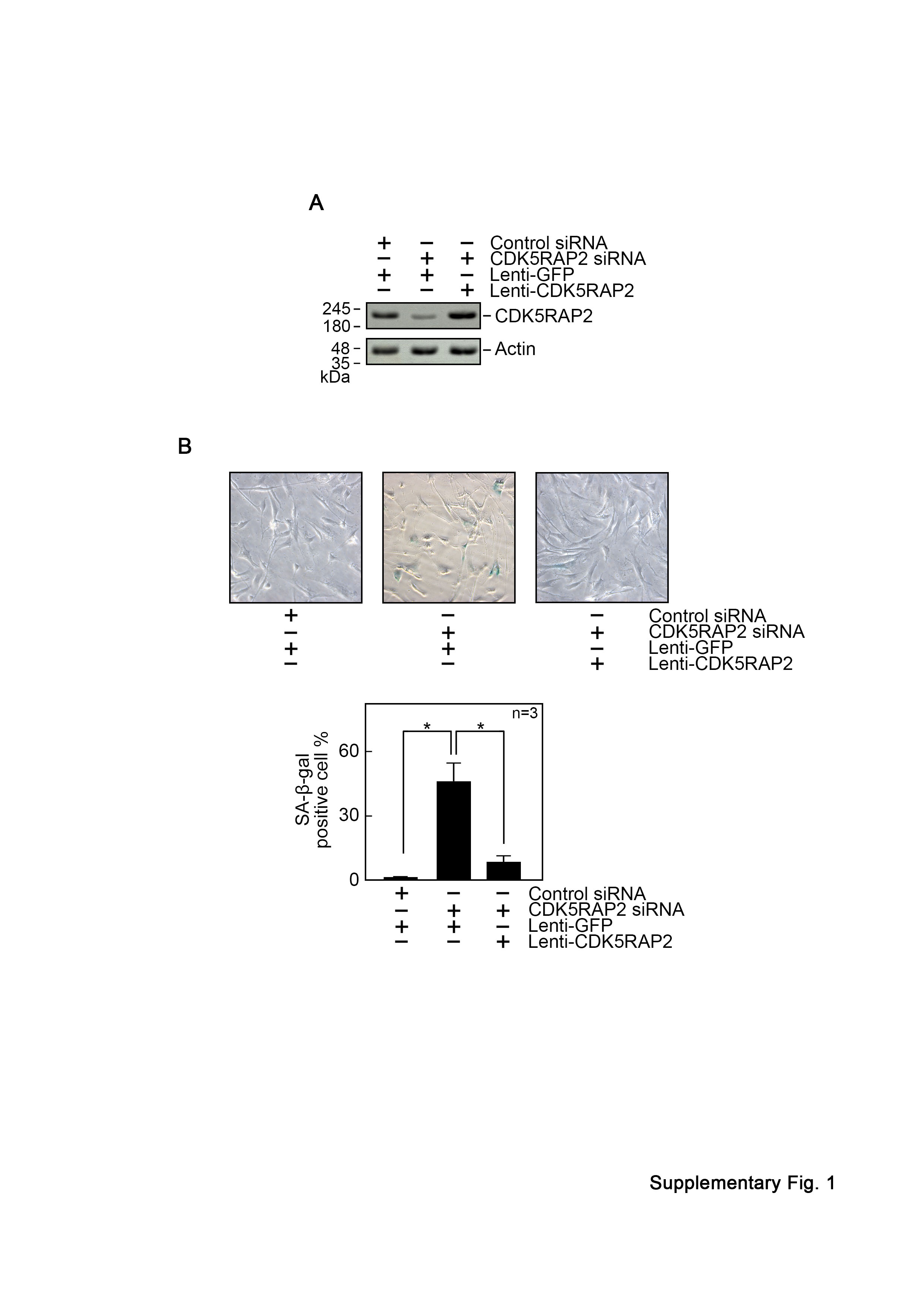

Supplement: Supplementary file 2 — Supplementary Figure 1 [file 41419_2021_4457_MOESM2_ESM.jpg]

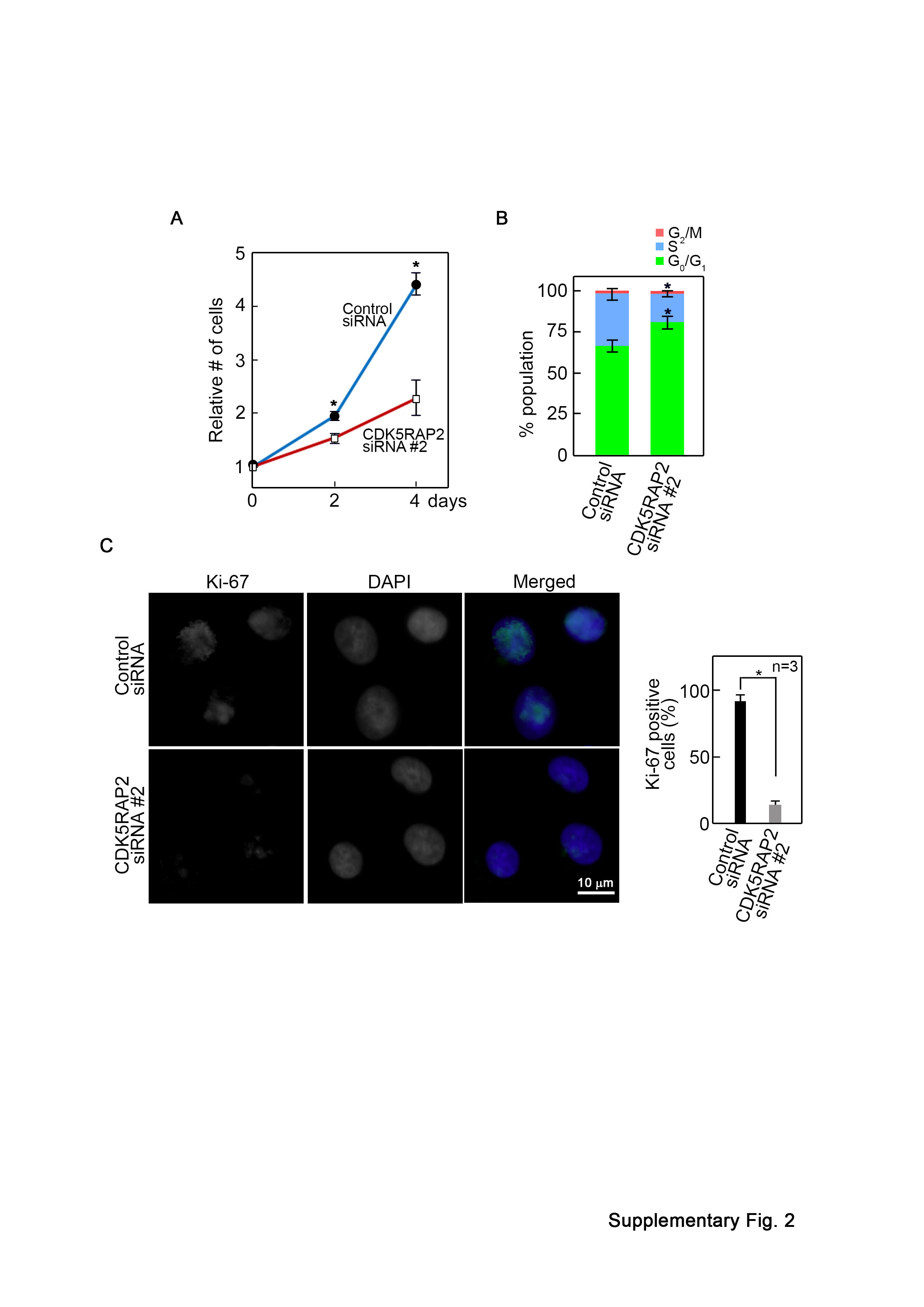

Supplement: Supplementary file 3 — Supplementary Figure 2 [file 41419_2021_4457_MOESM3_ESM.jpg]

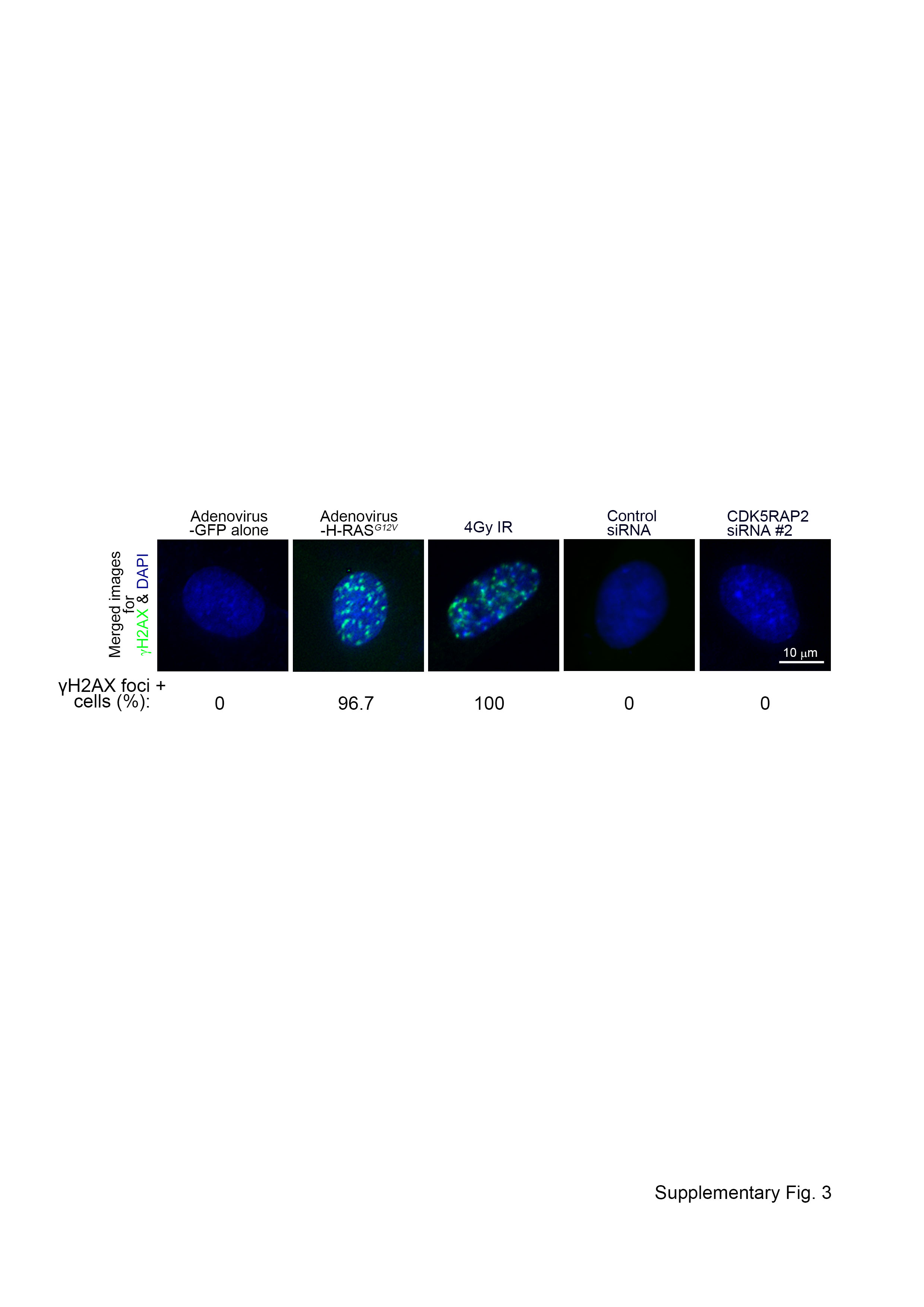

Supplement: Supplementary file 4 — Supplementary Figure 3 [file 41419_2021_4457_MOESM4_ESM.jpg]

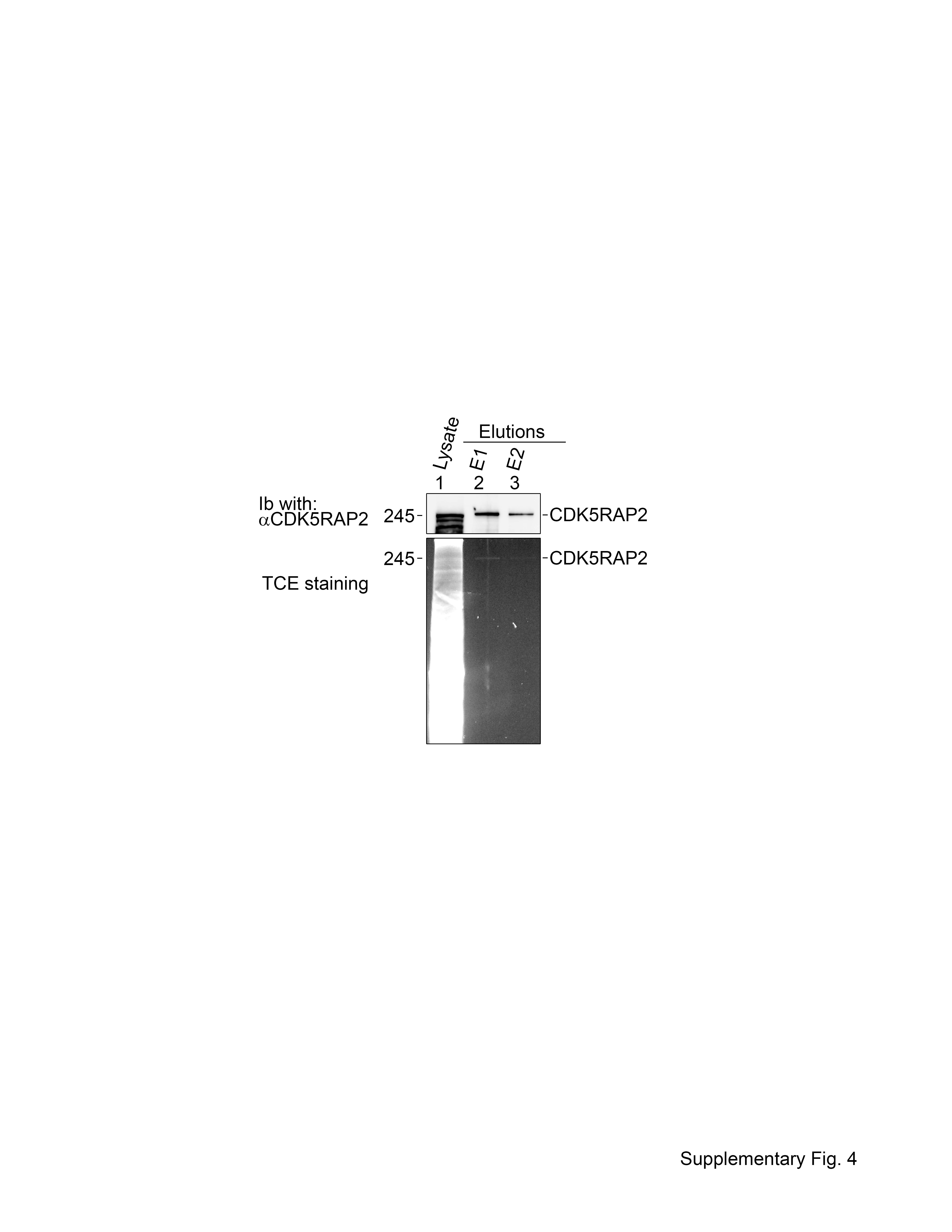

Supplement: Supplementary file 5 — Supplementary Figure 4 [file 41419_2021_4457_MOESM5_ESM.jpg]
